# Supplementary figures and images for: Gender Related Changes in Gene Expression Induced by Valproic Acid in A Mouse Model of Autism and the Correction by S-adenosyl Methionine. Does It Explain the Gender Differences in Autistic Like Behavior?
Source: Int J Mol Sci. 2019 Oct 24;20(21):5278. doi: 10.3390/ijms20215278 (PMC6862653; doi:10.3390/ijms20215278)

A

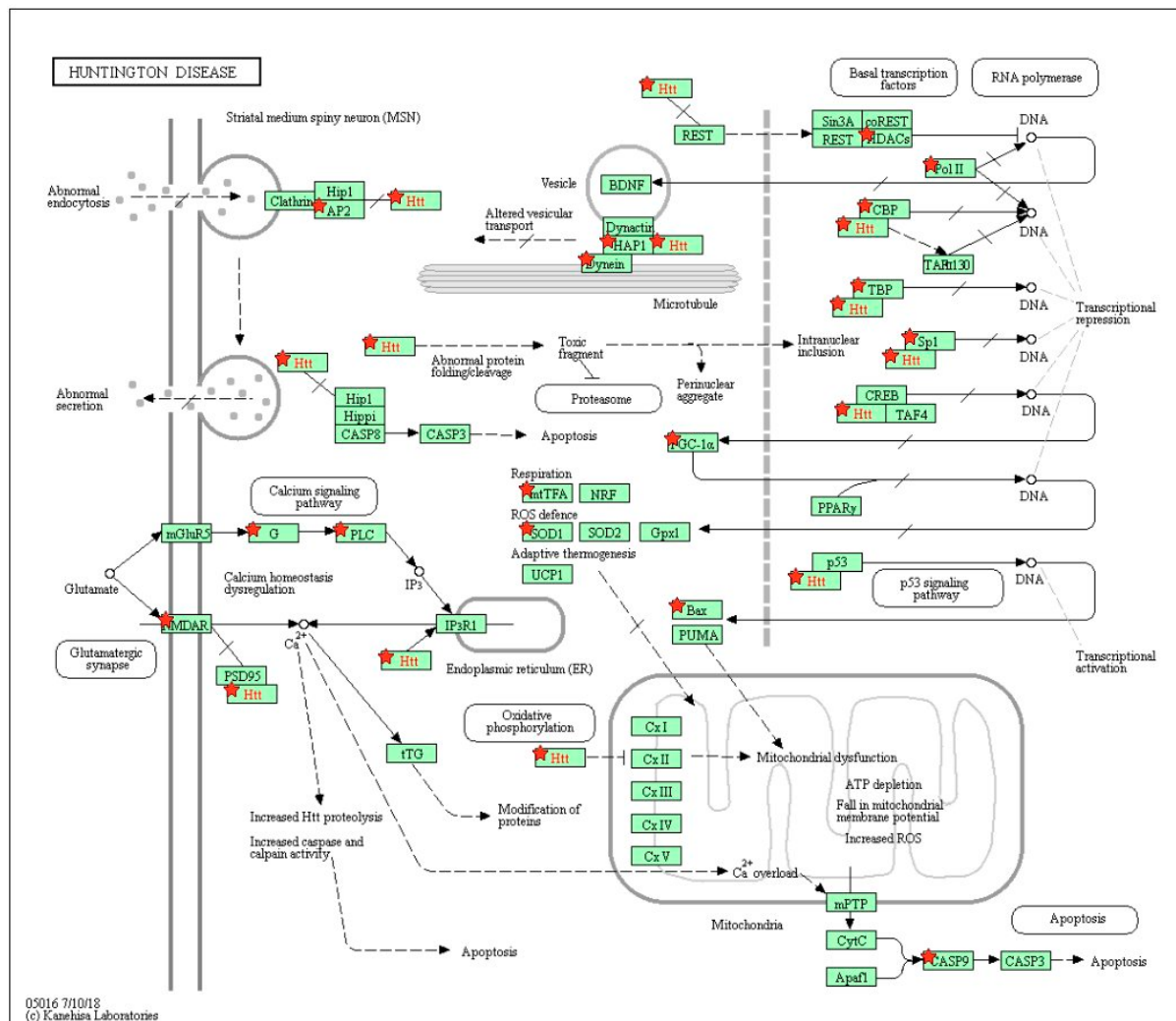

B

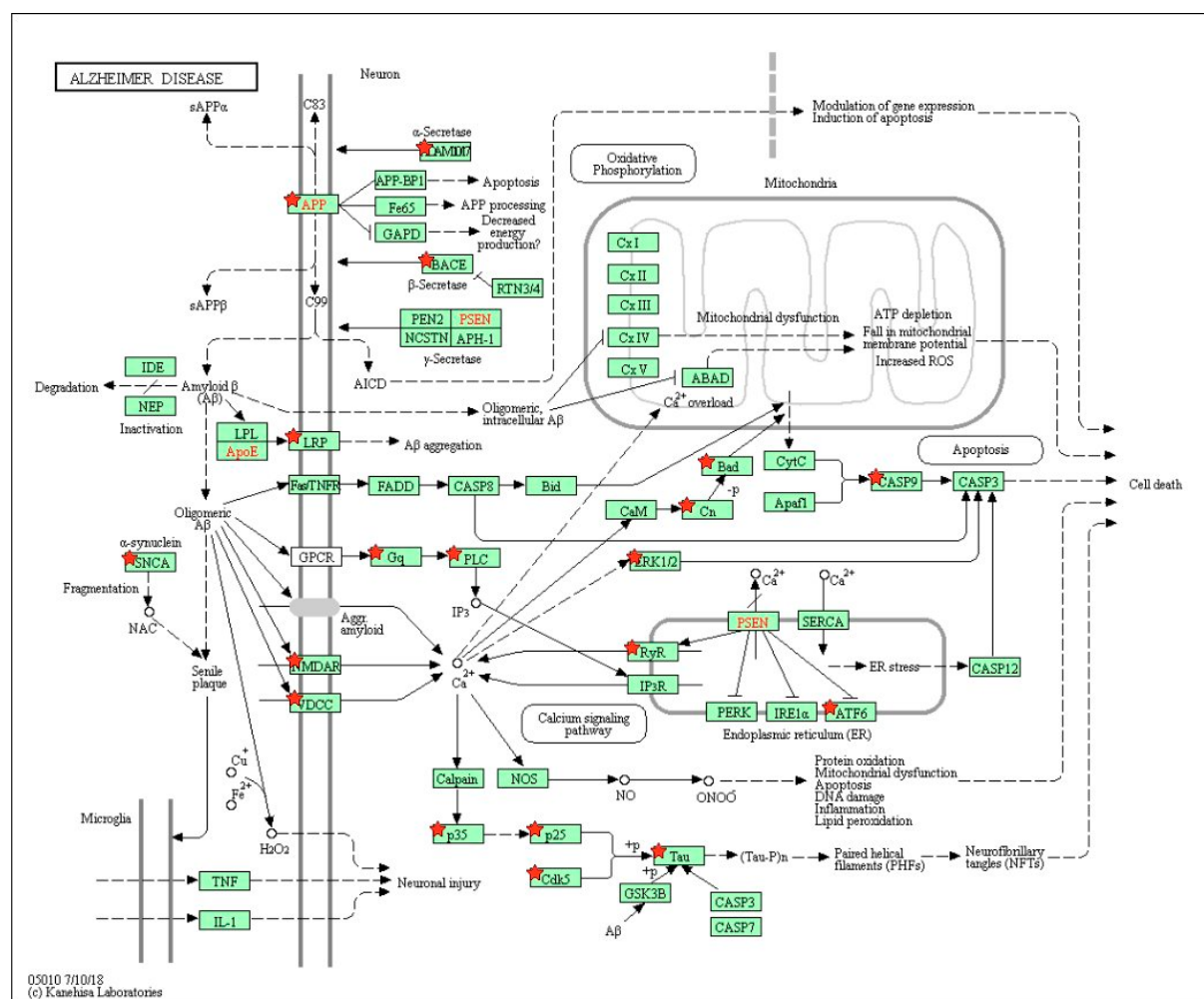

Supplement: Supplementary file 1 [file ijms-20-05278-s001.pdf]
